# The MYCN Protein in Health and Disease

**DOI:** 10.3390/genes8040113

**Published:** 2017-03-30

**Authors:** María Victoria Ruiz-Pérez, Aine Brigette Henley, Marie Arsenian-Henriksson

**Affiliations:** Department of Microbiology, Tumor and Cell Biology (MTC), Karolinska Institutet, SE-171 77 Stockholm, Sweden; aine.henley@ki.se (A.B.H.); marie.arsenian.henriksson@ki.se (M.A.H.)

**Keywords:** MYCN, embryonal development, childhood tumors, neuroblastoma, targeted therapy

## Abstract

*MYCN* is a member of the *MYC* family of proto-oncogenes. It encodes a transcription factor, MYCN, involved in the control of fundamental processes during embryonal development. The MYCN protein is situated downstream of several signaling pathways promoting cell growth, proliferation and metabolism of progenitor cells in different developing organs and tissues. Conversely, deregulated MYCN signaling supports the development of several different tumors, mainly with a childhood onset, including neuroblastoma, medulloblastoma, rhabdomyosarcoma and Wilms’ tumor, but it is also associated with some cancers occurring during adulthood such as prostate and lung cancer. In neuroblastoma, *MYCN*-amplification is the most consistent genetic aberration associated with poor prognosis and treatment failure. Targeting MYCN has been proposed as a therapeutic strategy for the treatment of these tumors and great efforts have allowed the development of direct and indirect MYCN inhibitors with potential clinical use.

## 1. Introduction

**Figure 1:**
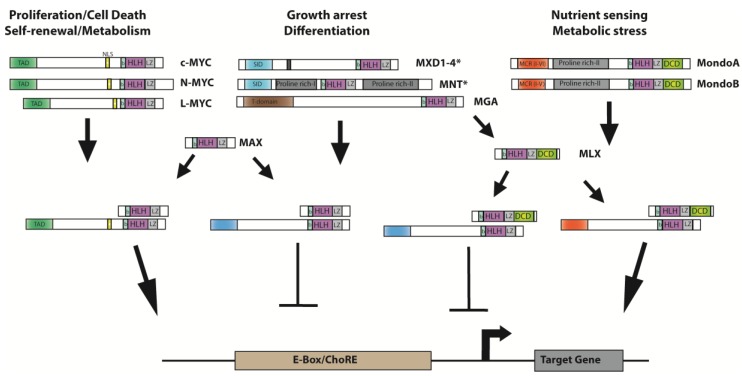
The MAX/MLX protein networks with domain organization of the members. The organization of the MAX and MLX network proteins with colored boxes indicating the functional domains. Each network reciprocal heterodimerization partner is indicated with its binding site. MAX: MYC associated factor X; MLX: MAX-like protein X; MGA: MAX gene-associated; MNT: MAX network transcription repressor; MXD: MAX dimerization protein; TAD: transcriptional activation domain; NLS: nuclear localization signal; b: basic region; HLH: helix loop helix; LZ: leucine zipper; SID: SID3-interacting domain; MCR: Mondo conserved region which contains six conserved regions creating a glucose sensing domain; DCD: dimerization and cytoplasmic localization domain; E-box: enhancer-box; ChoRE: carbohydrate response element. *MXD1, MXD4 and MNT bind to MLX while MXD2 and MXD3 do not.

The *MYC* proto-oncogene family includes three paralogs: *c-MYC*, *MYCN* and *MYCL* [1,2]. They are situated on different chromosomes (Table 1) and are expressed at specific times during development, but encode proteins with similar functional domains, including the trans-activating and DNA binding domains (Figure 1) [1]. These proteins, c-MYC, MYCN and MYCL (here together referred to as “MYC”), are transcription factors that belong to a larger class of proteins which contain a basic-region/helix-loop-helix/leucine-zipper (bHLHZip) important for protein dimerization and sequence-specific DNA binding [3]. The conserved transcriptional activation domain (TAD) is located in the N-terminus while the bHLHZip and nuclear localization sequences (NLS) are found in the C-terminus of the respective proteins (Figure 1). MYC is normally unstructured until dimerization with the MYC-associated protein X (MAX) to assist in DNA interaction [4,5]. The MYC family and its extended protein network is important in regulating several processes such as cell growth, differentiation and apoptosis [6]. Aberrant MYC regulation can lead to increased cell proliferation and is commonly observed in cancers [2].

MYCN is a 60–63 kDa protein with a short half-life of around 30 min [12] whose gene was first discovered in 1983 by Schwab et al. as a paralog to *c-MYC* [13]. Protein stability is controlled by the phosphorylation of specific residues. A first phosphorylation at Ser-62, mediated by cyclin-dependent kinase (Cdk1)-cyclin A/Cdk1-Cyclin B1 complexes, allows the recognition and phosphorylating activity of serine-threonine kinase glycogensynthase kinase 3β (GSK3β) on Thr-58 [14]. This modification leads to the ubiquitination and proteasomal degradation of MYCN [15,16]. The activation of the phosphoinositide 3-kinase/Protein kinase B (PI3K/AKT) axis is able to inhibit this process, since active AKT phosphorylates and inactivates GSK3β, thus leading to MYCN stabilization [16,17]. 

MYCN is part of the larger MYC/MAX protein network consisting of several bHLHZip regulators; the MYC family (c-MYC, MYCN and MYCL), the MXD (Max-dimerization partner) family (MXD1, MXD2, MXD3, MXD4), MAX network transcriptional repressor (MNT) and MAX gene associated (MGA) [18] (Figure 1). The extended members of the MYC transcriptional network all contain a conserved bHLHZip region along with individual conserved regions. The MXD family includes a SIN3-interacting domain (SID) region which directly interacts with the mSin3 corepressor associated with histone deacetylases (HDAC) [19]. Heterodimers of MYC and MAX form to bind to the E-box sequence CACGTG [20,21,22] and recruit histone acetyl transferases (HATs) and Tat-interactive protein 60 kDa (TIP60) to keep chromatin in an open configuration [3,20]. All MYC family paralogs and bHLHZip family members bind with MAX, and each hetero-complex has shared and unique sets of target genes [2]. The MYC/MAX heterodimers are essential for MYC function as the protein remains inactive until bound [2,23]. While MYC/MAX heterodimers promote proliferation through the recruitment of HATs, MXD/MAX represses transcription through HDAC activity [20]. Interestingly, overexpression of MXD can inhibit MYC controlled proliferation and can accumulate cells in the G0/G1 phase [18]. The opposite activities of MYC and MXD highlight the interconnected regulation between cell growth and cell death [20]. There is also a parallel network to MYC/MAX, which includes a MAX-like protein called MLX, that controls metabolism [24]. MLX is similar to MAX in that it contains a bHLHZip domain but additionally has a dimerization and cytoplasmic localization domain (DCD), which allows translocation from the nucleus to the cytoplasm following stress signals [18,25]. The MAX-like protein (MLX) binds to MXD1, MXD4, MNT as well as to two large bHLHZip proteins, MondoA and MondoB [18,24]. 

## 2. Cellular Regulation by MYCN

### 2.1. MYCN and the Cell Cycle

The role of MYCN in normal embryonic expansion is associated with cell proliferation and cell growth and is crucial for embryonic development [23] (see below). The cell cycle is a strictly regulated process under the control of the CDK family of serine/threonine kinases. These proteins act as heterodimers which contain two functionally important subunits, a catalytic subunit, CDK, and a regulatory subunit, cyclin, which activates the kinase activity of the protein [26]. The CDKs are constitutively expressed whereas the cyclins are intermittently induced to regulate the appropriate phase of the cell cycle and are degraded when not required [27,28]. *MYCN*-amplification has been shown to correlate with a failure of the cells to arrest in the G1 phase of the cell cycle [29], which could be due to promotion of cycle progression by MYCN through the upregulation of cyclin D2 [30]. 

The G1/S cell cycle checkpoint ensures correct progression to S phase, and MYCN inhibition has been shown to decrease efficacy of this transition [31,32]. This leads to a reduction of cells in the S phase and a resulting G1-accumulation coupled to repression of PI3K [32,33]. PI3K is important in cell growth, proliferation and metabolism [34]. As described above, inhibition of PI3K downregulates MYCN protein levels, lowering proliferation and leading to a reduction in S- and M phase cells [33]. Along with PI3K regulation, reduced MYCN is associated with changes in other cell cycle regulators such as the CDK inhibitor p27, E2 factor (E2F) and inhibitor of differentiation 2 (ID2). E2F is a key transcription factor in coordinating cell cycle progression and proliferation [32,35]. Inhibition of MYCN can decrease E2F levels, resulting in inactivation of genes involved in G1 phase and DNA replication [32,35]. ID2 is another regulator of cell proliferation and tumor progression that can inhibit the activity of neurogenic bHLH transcription factors [36]. 

A reduction of MYCN increases p27 levels, which in turn primarily results in inhibition of G1 CDKs [32,37]. Indeed, cell cycle progression is not only controlled through CDKs and cyclins, but also by growth factors and other proteins which are regulated by the MYC family. However, these effects on mitosis and its regulation are not fully understood.

In neuroblastoma with *MYCN-*amplification, copy number aberrations have been observed in genes regulating the G1 phase of the cell cycle [38]. Specifically, *CDK4* and *CDK6*, which are tumor promoting genes, have increased copy number gains or amplifications [38,39]. Targeting these two CDKs can cause tumor growth delay in vivo and their inhibition may constitute the basis for an novel therapies [39]. Importantly, the genetic regions most frequently lost or mutated in neuroblastoma patients contain three of the four classes of G1 cell cycle inhibitors [38]. It has also been shown that *MYCN*-amplified tumors have high E2F activity, which can result in increased activation of genes promoting the G1 phase [35,38]. In addition, the overexpression of ID2 correlates with poor outcome in neuroblastoma patients. Finally, inhibiting MYCN decreases ID2 activity, suggesting another therapeutic potential [36,40]. 

### 2.2. MYCN in Apoptosis and Cell Death

MYCN has both the ability to drive cell proliferation and growth, and can also promote apoptosis [41,42]. This is a programmed cell death mechanism that follows a specific set of biochemical and physical changes, including the activation of caspases and cell surface death receptors [43]. Apoptotic cell death is important during the development and maintenance of an organism as it ensures accurate embryogenesis and, later in life, assists in removing cells that are mutated or no longer needed [44]. 

Interestingly, MYCN has been suggested to have a dual role in apoptotic control due to acquired defects in molecular pathways [45,46]. It has been shown that MYCN is involved in the upregulation of the pro-apoptotic regulator phorbol-12-myristate-13-acetate-induced protein 1 (NOXA). Though MYCN does not directly induce apoptosis, it primes the cell to be more sensitive to cytotoxic drugs [41]. Inactivation of MYCN can lead to tumor regression through proliferation arrest and the induction of apoptosis [45,47]. The initiation of senescence and/or apoptosis is linked to its direct regulation of the adaptive immune response elements, programmed death-ligand 1 (PDL1) and CD47, which are decreased in correlation to reduced MYCN levels [47]. Remarkably, as previously mentioned, MYCN is involved in controlling the levels of chromatin acetylation in the cell through HATs and HDACs [20,48]. In addition, by using the HDAC inhibitor suberoylanilide hydroxamic acid (SAHA), MYCN protein levels were significantly reduced in *MYCN*-amplified cells, which was accompanied by increased apoptosis [48]. 

The p53 tumor suppressor protein is an important regulator of cell death, which responds to cell stress by inducing anti-proliferative mechanisms leading to apoptosis through activating several genes [49]. The levels of p53 are controlled by MDM2, which binds directly to p53 and regulates its stability through ubiquitination followed by proteasomal degradation [50]. However, p53 also activates MDM2 transcription leading to a feedback loop mechanism [50,51]. In certain contexts, an amplification of *MYCN* correlates with a deficiency in p53 expression allowing for aberrant cell growth [52]. Yet, the promotor region of the *p53* gene has an E-box element where MYCN can bind and activate its transcription [42,45]. Furthermore, MDM2 maintains cell proliferation by promoting MYCN expression without altering its stability [53]. On the other hand, MYCN can also increase MDM2 expression, exhibiting the feedback loop mechanism, which suggests a control of p53 activity by MYCN during cancer cell progression [51]. Moreover, inhibiting MDM2 activity in *MYCN*-amplified neuroblastoma sensitizes cells to chemotherapy [51]. Importantly, resistance to cell death has been linked to a combination of p53 loss of function and enhanced *MYCN-*expression [52]. 

Another regulator of apoptosis, *H-TWIST,* is often overexpressed in *MYCN*-amplified cancers [45]. *H-TWIST* is an anti-apoptotic onco-protein and its expression correlate strongly with MYCN levels [45,54]. H-TWIST controls the apoptotic response triggered by over-expressed MYCN as it contains a bHLHZip and can bind to MYC family members [54]. By inhibiting *TWIST* expression, the apoptotic response of *MYCN*-amplified neuroblastoma can be restored, providing evidence of a therapeutic target for drug development [55]. Interestingly, *H-TWIST* is also involved in attenuating p53 function [56].

### 2.3. MYCN and Metabolism 

Alterations in cellular metabolism are a crucial hallmark of cancer cells due to their need for rapid ATP production, increased biosynthesis of macromolecules and maintenance of cellular redox states [57]. Energy production is a tightly regulated process to ensure more energy is created than used [58]. In the presence of oxygen, glucose-derived pyruvate is converted to acetyl coenzyme A (acetyl-CoA) in the mitochondria and further processed through the tricarboxylic acid (TCA) cycle to produce more energy [59]. Most cancer cells have an altered metabolic phenotype, including the ‘Warburg Effect’, where cells primarily carry out glycolysis even in the presence of oxygen. This supplies an enhanced rapid production of ATP and carbons to support the biosynthesis of macromolecules by increased flux through branching metabolic pathways [60]. In this context, the TCA cycle has a less prominent role in energy production and, instead, is fueled by glutamine-derived carbons to function as an anaplerotic source of metabolites for biosynthetic processes [58]. Fatty acids provide important metabolic fuel and are also required for membrane synthesis, cell growth and proliferation [61]. Fatty acid oxidation is furthermore essential as a source of NADPH, which is used to inhibit oxidative stress. Targeting of this pathway can lead to mitochondrial dysfunction and increased generation of reactive oxygen species (ROS) [62,63,64].

The catabolism of glutamine helps to maintain pools of non-essential amino acids and to replenish the TCA cycle intermediates [65]. The importance of MYCN in cell proliferation shows its potential to support the enhanced demand of cancers cells for increased cell growth [66]. Tumors with an amplified *MYCN* gene increase cell growth through enhanced glutamine transport and glutamate metabolism [66,67]. When *MYCN*-amplified neuroblastoma cells were deprived of glutamine, the TCA cycle intermediates were depleted and cells underwent cell death [68,69]. This suggests that glutamine metabolism is essential in this context and is a regulator of key biosynthetic activities which are regulated by MYCN in a negative feedback loop mechanism [67,68]. Moreover, increased glycolysis and uptake of glucose is related to MYCN levels, thus suggesting a function via the “Warburg Effect” [5,70]. Furthermore, MYCN has been shown to increase glycolysis and glutaminolysis by stimulating mitochondrial biogenesis and function [64,67]. However, some studies have shown that there is little or no correlation between *MYCN-*amplification and the “Warburg Effect”, and this has been suggested to be a resistance mechanism to chemotherapy [71,72]. In addition, restoration of p53 in *MYCN*-amplified neuroblastoma increased sensitivity to chemotherapy in 50% of the tumors, suggesting resistance acquisition. Prior to this restoration, the tumors had increased glutathione S-transferase activity and restoring p53 and depleting glutathione re-sensitized the neuroblastoma tumors to chemotherapy [52]. This provides evidence into how complex the MYCN network is when evaluating its potential as a therapeutic target. 

Moreover, MYCN has been shown to not only increase glycolysis but also to stimulate mitochondrial biogenesis and β-oxidation [64]. Upon pharmacological MYCN inhibition, the mitochondria become shorter and form donut-shaped structures which have been linked to a cell protective mechanism [64,73,74,75]. Our group has demonstrated that MYCN inhibition leads to an increase in neutral cytoplasmic lipid droplets due to inhibition of mitochondrial respiratory complexes and β-oxidation [64]. Together, this shows that MYCN expression reprograms neuroblastoma metabolism.

Another interesting alteration to metabolism due to *MYCN*-amplification is the disruption of the cellular circadian molecular clock. This clock regulates a rhythmic gene expression for metabolism and it has been shown that glucose metabolism oscillation is disrupted by MYCN activity [76]. Although the underlying mechanism was not established, the oscillation of regulatory pathways is a novel control process that should be highlighted when studying metabolic regulators such as MYCN [76,77].

## 3. MYCN and Embryonic Tumors

### 3.1. MYCN, Embryonic Development and Pluripotency

As mentioned, MYCN has an important role during development, especially in the nervous system, with a strict spatial and temporal expression pattern. During mouse embryogenesis, *Mycn* is strongly expressed in gastrulating cells, and decreases afterwards in most tissues except in the central and peripheral nervous system, in the cranial and spinal ganglia and in the heart. Expression can also be detected in the developing lungs, kidney and gut [78]. In newborn mice, high levels of *Mycn* are found in the brain, the kidney and the intestine, but they progressively decrease while progressing into adulthood, with weak expression remaining in the adult brain, testis and heart [79]. During weeks 12–24 of gestation, high *MYCN* expression is found in undifferentiated neural cells in the brain, the retina, and in neuroepithelial cells in the lungs of human fetuses [80,81]. In another study, human fetuses between 16 and 19 weeks showed high *MYCN* mRNA levels in the differentiating epithelial mesenchyme in the kidney, and throughout the brain, especially in the intermediate zone between the subependymal and the cortical layers [82].

Homozygous null *Mycn* mice die between 10.5 and 12.5 days of gestation, showing multiple defects in organ development, especially in the heart and in the cranial and spinal ganglia, with a pattern more consistent with reduced proliferation than with impaired differentiation [83,84]. Heterozygous *Mycn* null mice only show a reduced survival, but no other observable phenotype [78]. In wildtype mice, *Mycn* is expressed throughout the developing neuroepithelium, with expression peaking in the neural tube, sensory neural-crest derived structures, forebrain and hindbrain [78]. In the null embryos, those normally *Mycn* high-expressing tissues showed structural deficiencies, reduced size and mitotic rate with fewer neurons [78,84]. Conditional knock-out of *Mycn* in neuronal progenitor cells leads to a two-fold decrease in brain mass, specially affecting the cerebellum and cortex, mainly mediated by reduced proliferation and enhanced differentiation, without evident effects on apoptosis [85]. Overexpression of *Mycn* in Japanese quail neural crest cells transferred into chicken embryos results in increased ventral migration and neuronal differentiation [86]. Conditional overexpression of the human *MYCN* gene under the rat tyrosine hydroxylase (TH) promoter in the TH-*MYCN* mouse model (see below) prevents the commitment of nestin positive sympathetic progenitors into the glial lineage, instead promoting the proliferation of paired-like homeobox 2B (Phox2B) positive neural progenitors and preventing their terminal differentiation [87]. Increased proliferation, apoptosis and commitment into the neural lineage were also consequences of overexpressing *Mycn* in mouse sympathoadrenal progenitor cells in vitro [88]. 

Importantly, MYCN has been more recently associated with the acquisition and maintenance of pluripotency. Exogenous MYCN expression, although not indispensable, is able to promote somatic cell reprogramming into induced pluripotent stem cells (iPSCs) [89]. MYCN promotes the expression of pluripotency associated genes in neural stem cells (NSCs) in vitro [90]. Both MYCN and c-MYC are involved in the proliferation, differentiation and survival of hematopoietic stem cells (HSCs) [91], and MYCN is in addition essential for the proliferation of cerebellum progenitor cells [92]. Deletion of endogenous *MYCN* and *c-MYC* in iPSCs and embryonic stem cells (ESCs) limits self-renewal capacity, pluripotency and survival, followed by induction of differentiation [93,94]. The potential involvement of MYCN in both stemness and proliferation may explain why the derregulated expression of the gene is involved in the development of a variety of human tumors and diseases (Table 2).

### 3.2. Neuroblastoma

During embryonal development, the neural crest is formed in the most dorsal part of the neural tube, in the structure that will become the brain and the spinal cord. The multipotent neural crest cells are able to undergo epithelial to mesenchymal transition and migrate to many body locations. Soon after the initiation of the migratory process, neural crest cells commit to progressively restricted cell lineages, finally differentiating into a variety of cell types, including the peripheral nervous system (neurons, glial cells, and Schwann cells), endocrine and paraendocrine cells, melanocytes in the epidermis, craniofacial cartilage, bone and connective tissue [95,96]. During this process, cell fate seems to be regulated by both positional signals and extracellular ligands. After arriving at their final location, the cells undergo terminal differentiation or apoptosis. A subgroup of neural crest cells situated in the trunk region of the neural tube will form the sympathoadrenal lineage, including sympathetic neurons and chromaffin cells [97,98]. The balance between cell proliferation, cell death, migration and differentiation is crucial during this sequence of events. Several mitogenic signals strictly control cell growth, and changes in the availability of those mitogens determine cell cycle exit and initiation of terminal differentiation. 

Pediatric neuroendocrine tumors, such as neuroblastoma, which arise during embryonal development, originate due to mistakes occurring during this complex process [99]. It has been suggested that embryonic tumors initiated from proliferating progenitor cells unable to differentiate, thus explaining why many childhood tumors have shorter latency periods and fewer genetic aberrations than adult tumors [100]. It is widely accepted that neuroblastoma derives from the sympathoadrenal lineage or its early neural crest-derived precursors, as evidenced from its common appearance in the adrenal gland medulla and in the paraspinal sympathetic ganglia [101]. 

Neuroblastoma is the most common solid tumor outside the brain, accounting for about 6% of all childhood cancers, with an incidence of 1/70,000 in children younger than 15 years. It has very heterogeneous clinical presentations and prognosis [102]. According to the International Neuroblastoma Staging System (INSS), stages 1 to 3 include localized tumors, while stage 4 groups patients with metastases. Intriguingly, neuroblastoma 4S (4 special) patients, infants with metastases in skin, liver and bone marrow, are characterized by the striking natural involution and regression of the tumors, frequently after minimal or no medical intervention [103,104]. Spontaneous regression also occurs in some patients affected by other neuroblastoma stages [105,106]. Several hypotheses have been proposed to explain how this regression occurs, including delayed activation of the developmental-related apoptotic program, immune response and loss of telomerase activity [103,107]. In situ neuroblastoma is described as microscopic adrenal tumors cytologically identical to neuroblastoma, with an incidence higher than that predicted from that of clinical disease, suggesting that only a small proportion of those microscopic neuroblastoma-like lesions develops into apparent tumors [108]. It has been proposed that those lesions are indeed remnants of normal sympathoadrenal development, containing cells which could eventually give rise to neuroblastoma [107]. 

This evidence supports the concept that neuroblastoma arises as a consequence of imperfect terminal differentiation, although the underlying mechanisms remain unclear. Several germline and sporadic mutations predisposing to neuroblastoma development have been identified, including *PHOX2B* [109,110], anaplastic lymphoma kinase (*ALK*) [111,112,113,114,115], polypeptide N-acetylgalactosaminyltransferase 14 (*GALNT14*) [116], and *MYCN* [13,117]. *MYCN-*amplification and -overexpression is considered the most robust prognostic factor for neuroblastoma patients, indicating poor outcome, even in localized disease, and it is used as a biomarker for patient stratification [101,118,119,120]. 

Neuroblastoma patients are stratified into low-, intermediate- and high-risk groups based on stage, age, *MYCN* status, histology and chromosomal ploidy. The event free survival of the high-risk patients remains around 40–50% even after intensive therapies and immunotherapy [121]. Moreover, these patients are often affected by severe side effects, including impaired growth and development, respiratory and cardiac problems, as well as difficulties in learning, problems with speech and behavioral disabilities. The treatment of high-risk patients consists of induction, consolidation, and maintenance phases. The latter aims to eradicate residual neuroblastoma cells after chemotherapy. Since these residual cells are usually highly resistant to conventional chemotherapy, the development of new therapies for these patients is a high priority [122]. 

Studies of the TH-*MYCN* mouse model have shown that MYCN is sufficient to drive neuroblastoma. In this model, human *MYCN* is overexpressed in the peripheral sympathetic nervous system under the TH promoter. TH expression is activated in migrating neural crest cells, and is the rate limiting step in the synthesis of catecholamines in the sympathetic ganglia and adrenal gland. In TH-*MYCN* mice, MYCN overexpression drives initiation and progression of spontaneous neuroblastoma-like tumors with high penetrance in the sympathetic ganglia [123,124]. These tumors are histologically and genetically very similar to aggressive undifferentiated human neuroblastoma, resembling the pathological and imaging characteristics, *MYCN* transgene amplification and tumor–stroma interactions [125]. Importantly, TH-*MYCN* mice treated with *MYCN* anti-sense RNA show reduced tumor incidence and tumor mass [126]. The Cre-conditional human *MYCN*-driven neuroblastoma mouse model, in which *MYCN* is conditionally expressed in dopamine β-hydroxylase-expressing cells in the neural crest, further supports the role of MYCN in neuroblastoma origination and development [127].

MYCN is critically involved in neuroblastoma tumor aggressiveness and resistance to therapy. *MYCN-*amplification is related to increased metastatic potential [120] and high MYC-signaling is correlated to poor prognosis, not only in the high-risk stratification group, but also in intermediate- or low-risk patients [128]. MYCN promotes cellular mechanisms linked to increased proliferation and evasion of apoptosis, which plays a major role in malignancy and chemoresistance. Exogenous expression of MYCN confers *MYCN* non-amplified neuroblastoma cells with an increased proliferative rate and tumorigenic capacity, mediated by enhanced autocrine growth-factor activity [129]. *MYCN-*amplified cell lines are resistant to apoptosis induction after DNA damage, due to reduced p53 signaling through the upregulation of MDM2 [130,131]. The members of the ATP-binding cassette (ABC) transporter family are able to expel drugs through the cell membrane and to confer multidrug resistance to cancer cells. It has been shown that MYCN upregulates the expression of several ABC transporters in neuroblastoma [132]. Importantly, induction of differentiation in neuroblastoma cell lines with retinoic acid leads to MYCN downregulation [133], while chemical or genetic downregulation of MYCN leads to neuroblastoma differentiation [64]. The presence of MYCN is required to complete the differentiation process of neuroblastoma cells treated with retinoic acid, since an increase of expression as an early event followed by a later reduction is indispensable to fulfill the differentiation process [133,134]. The control of cell metabolism by MYCN could also contribute to neuroblastoma malignancy. As mentioned above, MYCN downregulation induces accumulation of cytoplasmic lipid droplets, due to downregulation of mitochondrial ß-oxidation, accompanied by neural differentiation [64]. Furthermore, inhibition of glycolysis downregulates MYCN protein levels, leading to neuroblastoma growth arrest and cell death [135]. MondoA, a member of the MYC transcriptional network involved in metabolic control (see Figure 1) [18], interacts with MYCN to promote neuroblastoma cell survival through the upregulation of metabolic pathways [24]. 

### 3.3. Wilms’ Tumor

Wilms’ tumor, or nephroblastoma, is the most common childhood renal neoplasm with approximately one child affected per 10,000 worldwide, and accounts for 90% of renal tumors and 6% of all childhood cancers [136,137]. It arises as a consequence of impaired kidney differentiation [138]. Anaplastic tumor histology is a significant prognostic factor for poor outcome and is associated with *MYCN* copy gain [139]. Anaplasia is defined as a three-fold nuclear enlargement, hyperchromasia and abnormal mitotic figures [136]. Overexpression of MYCN is common in Wilms’ tumor even in the absence of increased copy number of the gene [136,139,140]. Though a recurrent copy gain on 2p24.3 has been observed which encompasses the *MYCN* locus [140]. Amplification of the *MYCN* gene results in increased transcription, which in turn leads to dysregulation of cellular process [140,141]. Some reports have suggested that MYCN function is potentiated by several mechanisms. Copy increase of *MYCN* accounts for 12.7% of tumors overall and in 30.4% of diffuse anaplastic Wilms’ tumors [140]. Point mutation at c.131C > T accounts for 3.8% of Wilms’ tumors and this mutation has also been observed in neuroblastoma with presumed gain of function [140,142]. *MYCN*-amplification dysregulates the cell cycle progress leading to relapse and poor survival [140].

### 3.4. Retinoblastoma

Retinoblastoma, an embryonic neoplasm of retinal origin, was initially thought to be caused exclusively by the loss of function of the retinoblastoma (*RB1*) gene, but *MYCN*-amplification has also been reported to initiate the disease [143,144,145]. It is the most common retinal cancer in children with approximately one incidence per 17,000 births and of these 40% are considered heritable [144,146]. Based upon the ‘two-hit theory’ (known as the Knudson hypothesis), retinoblastoma originally paved the avenue leading to better understanding of tumor-suppressor genes, which was confirmed by the discovery of the *RB1* gene [144,147]. Interestingly, MYCN expressing retinoblastomas are larger than *RB1*-only mutated retinoblastomas of the equivalent age [144]. Recent research show that some retinoblastomas with no evidence of *RB1* mutations carry an amplification of the *MYCN* oncogene [144]. Additional evidence has shown that *MYCN-*amplification is controlled by MDM2 through a p53-independent mechanism [53]. MDM2 increases *MYCN* transcription and translation and thus sustain the high levels of MYCN essential for increased retinoblastoma proliferation [53]. Conversely, rapid retinoblastoma cell death occurs when *MYCN* is knocked out [144]. Together, findings strongly indicate the therapeutic potential of *MYCN* gene in retinoblastoma. 

### 3.5. Medulloblastoma

Medulloblastoma is a childhood cancer where approximately 10% of cases are linked to the *MYCN* oncogene [33,148,149]. It is the most common malignant neuroectodermal tumor originating from the brain with an annual incidence rate of 6.8 cases/million children in Europe [33,150,151,152]. Medulloblastoma is classified into four major subgroups based on copy number variation and transcription profile [33,150]. *MYCN*-amplification has been observed in all four of the groups and is correlated with poor prognosis [150]. MYCN is the only member of the MYC family that is expressed in the proliferative zone of the cerebellum and it is regulated by MXD3 (see Figure 1) [20,153]. Upregulation of MYCN has also been observed in proliferating neural precursor cells in medulloblastoma [153]. As discussed above, MYCN is important in cell cycle progression and apoptosis and it is the dysfunction of these processes due to amplification of the *MYCN* gene that contributes to aggressive medulloblastoma [151,153]. 

### 3.6. Rhabdomyosarcoma

Rhabdomyosarcoma is a pediatric tumor of the mesoderm that comprises approximately 5% of solid childhood tumors and 55% of soft tissue sarcoma in children and adolescents [154,155,156]. This tumor is associated with the skeletal muscle lineage and is categorized into embryonal rhabdomyosarcoma (ERMS) and alveolar rhabdomyosarcoma (ARMS) [156,157]. *MYCN*-amplification has been observed in ARMS, with 25% of all cases showing a 5–20-fold amplification, and in 16% of ERMS [155,156,158]. *MYCN*-amplification is not the only critical event for aberrant cell growth of rhabdomyosarcoma, as copy number can influence cell growth. Interestingly, while other tissues and tumor type cell lines downregulate *MYCN* mRNA levels post-transcriptionally, in rhabdomyosarcoma, as in neuroblastoma, MYCN is expressed at both mRNA and protein level [157]. Amplification of *MYCN* is also associated with copy number gain [158,159]. ARMS patients with high levels of MYCN are prone to relapse and have a worse survival than those with lower MYCN expression [158]. The copy number gain and overexpression of MYCN are associated with a poor prognosis in ARMS patients and thus targeting MYCN as a novel therapy could be of relevance [158,159]. Initial reports of the inhibition of MYCN in rhabdomyosarcoma cells have shown reduced cell proliferation, induction of myogenesis and a rescue of p53 regulation [159]. The restoration of p53 has been linked to tumor regression and due to apoptosis and cell growth suppression [160]. The inhibition of cell growth by inhibiting MYCN shows promising therapeutic potential for rhabdomyosarcoma as well as other *MYCN*-amplified cancers. 

## 4. Other MYCN-Related Diseases

### 4.1. Feingold Syndrome

Feingold syndrome is an autosomal dominant disorder which results in digital anomalies, learning disabilities, microcephaly and short stature [161,162]. Since this syndrome is related to developmental aberrations, *MYCN*-amplification has been suggested to be a critical factor in dysfunctional development [163]. As mentioned previously, MYCN is normally only expressed during development and is critical in cell cycle progression [23,26]. It is important in the expression of forelimb mesenchyme stages and its dysregulation is consistent with distal bone malformations [161]. Interestingly, loss of function, deletions or heterozygous *MYCN* mutations can also cause Feingold syndrome [161,164]. Consistently, mutations in *MYCN* accounted for 50% of cases in Cognet’s study into Feingold syndrome. Specifically, the authors showed that syndactyly of toes 4 and 5 were more common in patients with *MYCN* mutations confirming the potent role of MYCN dysregulation in development is a crucial causative factor for Feingold syndrome [161].

### 4.2. Prostate Cancer

Prostate cancer is one of the most common cancers, with 57% heritability [165]. Neuroendocrine prostate cancer is a highly aggressive tumor arising in the later stages of the disease [166]. Focal neuroendocrine differentiation is present in 10% to 100% of prostate cancer which increases with progression [166,167]. Genetic instability has been linked to MYCN and more specifically 14% of all prostate cancers have *MYCN*-amplifications [168]. MYCN has been shown to repress androgen receptor signaling in prostate cancer and can redirect the binding of the transcription factor enhancer of zeste homolog 2 (EZH2) to the *MYCN* promoter resulting in gene silencing [169]. Furthermore, MYCN and Aurora Kinase A (AURKA) have higher expression in late stage neuroendocrine prostate cancer than other prostate cancer types and they have been suggested to cooperate in inducing differentiation [166]. A positive feedback loop has been described whereby AURKA induces MYCN and it has therefore been suggested that this cooperation leads to the prostate cancer phenotype [15,167]. AURKA has also been shown to interact with MYCN in neuroblastoma and may play important roles also in other tumors [167]. Inhibitors of AURKA have been suggested as a therapeutic option for *MYCN*-amplified cancers as they induce degradation of the MYCN protein (see below) [170]. Moreover, inhibition of EZH2 may provide synergy with the AURKA inhibitors, furthering the clinical outcome [169]. 

### 4.3. Basal Cell Carcinoma

Basal cell carcinoma accounts for 75% of all skin cancers and is the most frequent neoplasm in humans, however with a relatively low mortality rate [171]. Copy number gains and amplification of *MYCN* have been identified in basal cell carcinomas and, as reported in other cancers, this is associated with aberrant cell growth [171]. In basal cell carcinoma, increased sonic hedgehog (SHH) has been linked to increased MYCN [171,172]. SHH has been related to both basal cell carcinoma and medulloblastoma which, in some forms, have amplification of the *MYCN* locus [33,173]. The relationship between these two regulators could be important to study further for therapeutic purposes. Targeting Smoothened, the positive regulator of the hedgehog pathway, resulted in tumor regression in medulloblastoma patients with SHH mutations [173,174]. The collaboration of SHH and MYCN may be pivotal in both medulloblastoma and basal cell carcinoma regulation, and further insights are necessary for the identification of potential novel therapeutics. 

### 4.4. Leukemia

Both chronic lymphocytic leukemia (CLL) and acute lymphoblastic leukemia (ALL) have been associated with *MYCN*-amplification [175,176]. CLL is a disease characterized by the accumulation of mature lymphocytes [175]. Copy number gain and amplification of *MYCN* have been documented in CLL [177]. Interestingly, *MYCN*-amplification was also predominantly evident in a patient with a hypermutation of *p53* [175]. This is interesting due to the functional interaction between p53 and MYCN in rhabdomyosarcoma, in which inhibition of MYCN leads to a restoration of p53 [159]. Interestingly, the relationship between p53 and MYCN has been linked to suppression of Sirtuin1 (SIRT1), indicating a role in epigenetic modification [52,178]. Another leukemia, T-cell ALL (T-ALL) is associated with poor prognosis and accounts for 15% of ALL cases [176,179]. *MYCN-*amplification is evident in 50% of T-ALL patients and its inhibition results in reduced cell viability [176]. Increased MYCN expression has also been detected in acute myeloid leukemia and in ALL [180,181]. Treatment of T-ALL cells with a peptide nucleic acid specific for MYCN showed a significant inhibition of cell growth and this complementary therapy could thus have great therapeutic potential [176]. 

### 4.5. Lung Cancer

As mentioned previously, MYCN expression is frequently high in the developing lung epithelium and is important for epithelial mesenchymal interactions [6,182]. When the gene is mutated to a null allele or a conditional knock out in utero, there is a reduction in lung growth and reduced branching [6]. Lung cancer is one of the most common cancers in the world [182,183]. Along with *p53* and *RB1* genomic alterations, amplification of the *MYC* family is observed in 20%–40% of small cell lung cancer (SCLC) cases [167,182,184,185]. Lung cancer is unique in that all three MYC family members, c-MYC, MYCN and MYCL, are implicated in this disease and have been shown to activate or repress different subset of genes in SCLC cells [186]. *MYCN* has a copy number gain greater than 4 and amplified expression is observed in 6% of all cases [184]. As with prostate cancer, AURKA has been linked to the disease but its inhibition does not alter MYCN levels, suggesting a difference in activity in *MYCN*-amplified non-small lung cancer cells (NSCLC) compared to *MYCN*-amplified neuroblastoma and prostate cancer [184]. Other targets of the MYCN pathway such as SOX2, p53, miR-34a and miR-9, have been proposed to affect the poor prognosis of NSCLC [182,183,187]. SOX2, a transcription factor involved in differentiation, proliferation and apoptosis, has been shown to be frequently amplified in NSCLC. High expression of MYCN correlates with SOX2 expression, suggesting that their activity is linked, but further investigation is required [183]. MYCN has been proposed to drive cell death in part through p53 [147,159,182]. p53-induced miR34a expression is associated with cisplatin sensitivity in NSCLC through the suppression of MYCN. MiR-34a induces apoptosis and is a direct target of p53 and by downregulating MYCN it can increase the chemotherapy potential in NSCLC [182]. In contrast, miR-9 is activated by *MYCN* and its amplification correlates with over expressed *MYCN*, advanced tumor grade and metastasis. This correlation has not only been shown in NSCLC but also in neuroblastoma. Importantly, miR-9 suppresses the expression of E-cadherin to promote invasiveness and angiogenesis [187]. The balance between the need for high MYCN levels during lung development and its aberrant high expression in lung cancer is not well characterized. Importantly, there is a great potential to integrate the inhibition of MYCN function to strategies for novel cancer treatments. 

## 5. MYCN as a Therapeutic Target

**Figure 2:**
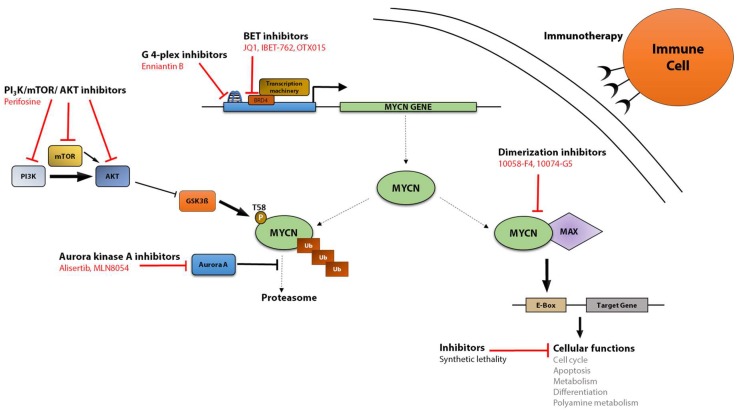
Strategies for direct and indirect targeting of MYCN**.** Different approaches have been developed to hinder MYCN activity, including inhibition of *MYCN* gene transcription, disruption of the dimerization with its transcriptional partner MAX, induction of MYCN protein destabilization, inhibition of MYCN-promoted cellular processes as well as immunotherapy. Specific inhibitors are highlighted in red. Solid lines indicate direct activity. Dashed lines indicate different protein status (inactive monomeric, active dimeric and degradation-targeted phosphorylated MYCN). PI3K: phosphoinositide 3-kinase; AKT: protein kinase B; GSK3ß: glycogen synthase kinase 3 beta; mTOR, mammalian target of rapamycin; BRD, bromodomain; BET, bromodomain and extra terminal domain; Ub: ubiquitin.

As reviewed above, MYCN is a complex regulator that has several seemingly contradicting functions, as it is, for instance, involved in both cell cycle regulation and apoptosis [45], depending not only on the state of the cell or the level of amplification and copy number gain [45,188]. Heterogeneity of MYCN has been documented recently to vary from tumor sites, during cancer progression and even following treatment [188]. This highlights the need to fully understand MYCN and its complex relationship to other proteins as well as its targets within the cell. As described above and summarized in Table 2, *MYCN* genetic aberrations are involved in the development of a wide variety of diseases. Many malignancies would benefit from improved MYCN-targeting approaches with therapeutic purposes. Several strategies have been developed to date to target MYCN at the transcriptional or protein level, including protein stability and dimerization, immune therapy and synthetic lethality (Figure 2). These approaches have shown different efficacy and success, some of them providing very promising results. 

### 5.1. Targeting MYCN Transcription

Guanine quadruplexes (G-quadruplexes) are G-rich DNA strands that can fold into non-canonical secondary structures consisting of four-stranded helixes [189]. As is the case for *c-MYC*, a G-quadruplex has been found close to the *MYCN* promoter region [190]. It has been suggested that the G-quadruplex at the *c-MYC* promoter could be involved in the positive control of *MYC* expression and that small molecules with high affinity for this structure could have the ability to inhibit the gene transcription [191]. In fact, several small molecule ligands for the *c-MYC* G-quadruplex have been described with promising preclinical results that prompted some of them to reach Phase II clinical trials [192,193]. However, the clinical responses were not as strong as expected. There are only few publications investigating MYCN G-quadruplex ligands, but enniatin B, a fungal metabolite with antibiotic roles, has been described to selectively bind the *MYCN* G-quadruplex in comparison to B-cell lymphoma 2 (*Bcl2*), c-myeloblatosis (*c-myb*), *c-myc,* cell adhesion molecule L1 like (*Chl1*), mast/stem cell growth factor receptor (*C-kit*) and Tel23 G-quadruplexes [194].

The bromodomain and extra terminal domain (BET) proteins are a subgroup of the bromodomain-(BRD) containing protein family [195]. These are evolutionary conserved domains forming hydrophobic pockets that specifically recognize acetylation marks in lysine residues [196]. Lysine acetylation of histones is an epigenetic modification linked to an open chromatin structure and increased transcription [197]. BET proteins recognize specific marks in chromatin and interact with them to act as core elements for the recruitment of transcriptional regulatory complexes [198]. BRD4, a member of the BET family, is involved in the control of *MYC* transcription, and small molecule inhibitors targeting BET proteins, such as JQ1, are able to downregulate *MYC* expression [199]. *MYCN*-amplification was found to be correlated to sensitivity to the BET-inhibitor JQ1 in neuroblastoma cells, leading to impaired growth, increased apoptosis and downregulation of MYCN transcriptional program. Furthermore, BRD4 knockdown pheno-copied the JQ1 effects [200]. Another BET inhibitor, I-BET726, was shown to suppress MYCN as well as BCL-2 expression and accelerate apoptosis in neuroblastoma cell lines independently of *MYCN* copy number or expression level, and led to reduced tumor growth in vivo [201]. An additional BET inhibitor, OTX015, has also been shown to reduce neuroblastoma growth in vitro and in vivo through the inhibition of BRD4, which occupies not only *MYCN* promoter, but also enhancers of MYCN target genes [202]. 

### 5.2. Targeting MYCN Protein Stability

As described in the introduction, MYCN is a very short lived protein, with a half-life of around 30 min [203]. Its stability is controlled by phosphorylation, ubiquitination and proteasomal degradation. Chemical intervention with proteins involved in the control of MYCN stability may potentially have an impact on MYCN-driven tumors. AURKA is able to form a complex with MYCN and protect it from proteasomal degradation. Small molecule inhibitors of AURKA, such as MLN8054 and MLN8237 (Alisertib), have been described to reduce MYCN stability by increased degradation, to increase survival in an in vivo model of neuroblastoma [204], and to reduce neuroblastoma-induced endothelial cell proliferation [205]. However, not all AURKA inhibitors are able to affect MYCN stability, and it has been recently published that only those able to distort the kinase structure in a way incompatible with MYCN interaction have this effect [206]. Alisertib is currently in Phase II clinical trials, following promising response and progression-free survival in combination with conventional chemotherapy in refractory neuroblastoma patients in a Phase I study [207]. Inhibitors for the PI3K/mammalian target of rapamycin (mTOR)/AKT axis lead to increased GSK3ß activity and, thus, to increased MYCN protein degradation, inducing growth arrest and apoptosis in neuroblastoma cells both in vitro and in vivo [17,208,209,210,211]. The PI3K/AKT inhibitor perifosine has in clinical trials demonstrated the ability to prevent progression in high-risk neuroblastoma patients with refractory or resistant disease [212].

### 5.3. Targeting MYCN Dimerization and Transcriptional Activity

Inhibition of MYC interaction with its transcriptional partner MAX is a promising therapeutic approach to hinder its activity. However, the MYC dimerization domain is structurally characterized by inherent disorder [213], and the MYC-MAX crystal structure does not show obvious targetable amino acid residues or structural pockets [214], which makes it difficult to design small targeting molecules. However, high-throughput screening of a drug library has allowed the identification of several small molecule inhibitors targeting c-MYC dimerization with MAX and their binding to DNA [213,215,216]. Some of them, including 10058-F4 and 10074-G5, have, in addition, also been identified to target MYCN and to induce neuroblastoma cell differentiation and apoptosis [217,218]. The proteomic profiles of *MYCN*-amplified neuroblastoma cells treated with 10058-F4 or with specific *MYCN* short-hairpin RNA (shRNA) show similar affected Gene Ontology pathways and processes, where about half of the downregulated genes were previously characterized as MYC target genes [64]. Although these compounds show promising effects in vitro, there are only few studies showing efficacy in vivo. Among them, our group showed that 10058-F4 is able to induce increased survival in TH-*MYCN* mice, and the treatment delayed tumor growth in a *MYCN*-amplified neuroblastoma xenograft [64]. However, these chemical compounds have a low solubility and are quickly metabolized, leading to a short in vivo half-life as well as reduced concentration and efficacy in tumors [219,220], which makes them ineffective candidates for the clinic. Still, their molecular structures and mechanism of action are being used as the basis for the development of improved small molecule inhibitors of MYC [221] that could potentially also be used to target MYCN. 

### 5.4. MYCN-Based Immunotherapy

An appealing strategy to treat patients with tumors expressing high MYCN levels, including *MYCN*-amplified neuroblastoma, consist of developing immune therapies based on vaccination against MYCN protein as a tumor-associated antigen. Circulating T lymphocytes are able to recognize antigens exposed in the cell surface. These antigens consist of short peptides resulting from the degradation of endogenous or exogenous endocytosed proteins, which are transported to the cell membrane in association with human leukocyte antigen (HLA), forming the so-called major histocompatibility complex (MHC) [222]. As in the case of proteins normally expressed in healthy cells, the peptides derived from oncogenes in cancer cells may be exposed in this way, constituting tumor antigens. It has been shown that MYCN may act as a tumor-associated antigen, since MYCN-specific cytotoxic T-cells were detected in *MYCN*-amplified neuroblastoma patients [223]. Only a few studies have been published on MYCN-based immunotherapy, but they show promising results. Cytotoxic T lymphocytes generated using a MYCN-derived peptide containing a HLA-A1 binding motif were able to lyse HLA-matched, *MYCN*-amplified neuroblastoma tumor cells in vitro [223]. In a more recent study, immunocompetent MYCN-expressing syngeneic mice were immunized with a DNA vaccine derived from a *MYCN* minigene or full-length *MYCN* complementary DNA (cDNA). Intriguingly, both vaccines are able to reduce primary tumor growth through the induction of T cell-mediated response in vivo [224]. 

### 5.5. Indirect Targeting of MYCN

There are several indirect strategies to target MYCN-driven tumorigenesis. One possible approach consists of inhibiting the function of key target genes of MYCN involved in tumor-promoting processes. Like c-MYC, MYCN is involved in the control of metabolic processes. Targeting key enzymes involved in metabolic pathways, such as ornithine decarboxylase (ODC), which catalyzes the rate limiting step on polyamine biosynthesis, and hexokinase (the first step on glycolysis), which not only induces neuroblastoma cell death, but also reduces MYCN protein levels [135,225]. DFMO (2-(difluoromethyl)ornithine), an ODC inhibitor, extended tumor latency in TH-*MYCN* mice, and combination treatment of DFMO with another polyamine synthesis inhibitor, SAM486, protected 40% of the mice from developing neuroblastoma. Additionally, the combination of DFMO with classical chemotherapy increased the survival after tumor initiation [225]. Several Phase I clinical trials with DFMO in neuroblastoma patients have shown promising results [226,227]. Another strategy is based on targeting synthetic lethal interactions of MYCN. The term synthetic lethality refers to the response by which the joint deficiency in the expression of two or more genes will lead to increased cell mortality while the reduction of each single gene alone will not. Experimental screening of shRNA or RNA interference (RNAi) libraries has allowed the identification of MYCN synthetic lethal candidate genes involved in DNA repair, MYCN protein stability, metabolism and the cell cycle, such as AURKA, CDK1, CDK2, checkpoint kinase 1 (CHK1), adenosylhomocysteinase (AHCY), or Bloom Syndrome RecQ Like Helicase (BLM) [15,228,229,230], opening a door for combined therapy approaches against MYCN-expressing tumors.

## 6. Conclusions

MYCN plays crucial roles during embryonic development, being involved in the control of a large variety of cellular processes fundamental for organogenesis, such as regulation of pluripotency, cell cycle, apoptosis and metabolism. It is the ability to promote these various mechanisms which makes pathological MYCN activation able to initiate and support malignant phenotypes. Understanding of *how* those processes are controlled, *how* MYCN contributes to them, and *how* they can be targeted is fundamental in order to develop novel and efficient therapeutic strategies. Advances in the knowledge of the roles of MYCN during both physiological and pathological conditions, as well as in the development of small molecule inhibitors, will provide new possibilities to improve patient treatment. Targeting MYCN as an effective anticancer therapy seems today to be an objective that is closer to reality than ever. 

## Figures and Tables

**Table 1:** Location of the *MYC* family genes in the human and mouse genome.

	Chromosomal Location		
Gene	Human	Mouse	References
*MYCN/Mycn*	2p24.3	12-A3.B	[7,8]
*MYCL/Mycl*	1p34.2	4-D2.2	[8,9]
*MYC/c-myc*	8q24.21	15-D1	[10,11]

**Table 2:** Aberrations of *MYCN* in human disorders.

Disease	Onset	Primary Location	*MYCN* Aberration	% of Patients Showing *MYCN* Aberration
Neuroblastoma	Childhood	Peripheral nervous system	Amplification/overexpression	25%
Medulloblastoma	Childhood	Cerebellum	Amplification	7–10%
Retinoblastoma	Childhood	Retina	Amplification	2%
Rabdomyosarcoma (ARMS/ERMS)	Childhood	Skeletal muscle	Copy number gain/amplification/Increased stability	25% of ARMS 16% of ERMS
Wilms’ tumor	Childhood	Kidney	Copy number gain/Overexpression/mutation	13% 9% 4%
Feingold syndrome	Childhood	Physical and learning developmental disorders	Amplification/ deletion/loss of function	50%
Leukemia (CLL/ALL)	Childhood/adulthood	White blood cells	Copy number gain/amplification/Increased expression	7% of ALL 35% of CLL
Prostate cancer	Adulthood	Prostate	Amplification	14%
Basal cell carcinoma	Adulthood	Skin	Copy number gain/increased expression	18% 73%
Lung cancer	Adulthood	Lung	Copy number gain	6%

ARMS: Alveolar rhabdomyosarcoma; ERMS: Embryonal rhabdomyosarcoma; CLL: Chronic lymphocytic leukemia; ALL: Acute lymphoblastic leukemia.
